# Case of Poisoning by Arsenic, Successfully Treated

**Published:** 1821-12

**Authors:** Jos. Hume


### *Case of Poisoning by Arsenic, successfully treated.*
By Jos. Hume, Esq.

CONFORMABLY to the promise in my last letter,* I now communicate some further circumstances concerning the case of recovery from the poison of arsenic; which cure I still believe to have been accomplished, mainly, by a frequent exhibition of *carbonate of magnesia* and *opium*, especially during the commencement of the patient's sufferings.

There are several cases of recovery from this deleterious drug already before the public, in which great skill and a judicious variation of treatment with that of the symptoms are very conspicuous. In some of these instances, it must be observed, after the primary and specific effects on the constitution had been overcome and begun to decline, that a very different class of indications presented themselves to the practitioner's notice, requiring a total change in the management of the patient, and the employment of opposite remedies. Among other examples of this versatility of appearances, and the consequent deviation in the practice, I shall refer your readers to Dr. Roget's paper, published in the Medico-Chirurgical Transactions, vol. ii. and to Mr. Marshall's Remarks on Arsenic, quoted in my last communication; both of which I consider to be excellent authorities for the best instructions on similar occasions.

In this woman's case, the abatement of the specific signs,—such as the excessive retching; sensation of a burning heat in the fauces and other parts subservient to deglutition; fixed and severe pain about the præcordia and upper portion of the abdomen; together with frequent, but not effectual, evacuations from the bowels;—was succeeded by a train of very contrary symptoms. The principal of these were chilliness, with cold extremities; nausea, and slight efforts to vomit; a trifling head-ache; a peculiar feeling of smarting and pain at the termination of the rectum; an inclination to evacuate, but not at all of the nature of tenesmus; and some oppression in the chest, with difficulty of breathing.

The most urgent of these sequelæ were the two last, especially the dyspnœa; and this was presently relieved by tinctura hyoscyami, given every three hours, with the common saline mixture, solutio potassæ citratis. Each dose of the tincture did not exceed six minims, and this was continued for nearly two days, the breathing having by that time been perfectly restored. For the irritation in the rectum, I gave the patient two copious doses of oleum ricini; and this medicine, with those already described, constitute the whole that were required in the present case.

---

* Page 468.

To ascertain the quantity of arsenic which had been taken, I weighed the remaining portion in the paper, believing that a certain weight by avoirdupois had been purchased, and that the deficiency would indicate the dose. I also questioned the woman herself, as well as her mother, respecting every particular, so as to elicit the truth; and I found that the powder had been conveyed into the glass on the point of a common table-knife; that cold water was added, and the whole stirred well with a tea-spoon : this dose was then instantly swallowed; and, as the patient said, it had no other taste than a "gritty or sandy sensation among her teeth."

Taking every circumstance into consideration, I am confident that more than eighty grains were mixed in the water. However, from the very ponderous and insoluble nature of the white oxide of arsenic, especially in cold water, it is proper to make a fair allowance for that portion which must subside or adhere to the surface of the glass. I regret that I could not proceed in this part of the inquiry, for the mother of the patient was too officious to allow the glass to remain a moment for my inspection: it was well washed out, even before she called for my assistance.

Whether the mode of mixing the arsenic, and the state of the stomach on receiving it, can have any influence on the subsequent action of the poison, seem to be questions of some import and deserving at all times to be taken into the account. In the five cases related by Mr. Marshall, the powder was mixed in that species of food known by the name of *yeast-dumplings*, in which, to my knowledge, a considerable quantity of the arsenic was introduced; for I received the mere scrapings of the dish in which the paste had been kneaded, and of this every atom was loaded with the poison, and too palpable in *quantity* to require much trouble or nicety in the analysis.* The viscid nature of this paste would tend greatly to sheath and envelop the arsenic; and, consequently, when the stomach in such a case is excited, and vomiting ensues, there is a probability of

* The writer in the *Journal of Science and the Arts*, vol. iii. p. 37, has betrayed great ignorance of the case of Eliza Fenning. There was no doubt, even in the minds of the rabble that formed the "public disturbance" and "discontent," that arsenic was mixed in the food which this woman dressed for Mr. Turner's family; the learned judge, the jury, and the court, were perfectly satisfied of that part of the evidence; the conviction was complete; and the sentence, and its execution, were what the culprit well deserved. "Public disturbance" is seldom on the right side; I believe it is always prone to mischief. The unmerited attack upon the house of one of the best of men, the late venerable president of the Royal Society, in Soho-square, about seven years ago, is another instance of what "public disturbance or discontent" would accomplish, were it not opposed. The writer ought to have been very correct, and to have known that the question of doubt, and that by the *public disturbers* only, was, *who* put the poison into the food? Mr. Marshall's publication has, however, set this question at rest.

much of the powder being ejected out of the system, on every effort made by that organ to relieve itself.

I have already described the supper which my patient had eaten, just before she took the poison;—that this meal was more of a solid than liquid or porous nature; and I have also mentioned that she was at the time in a high state of intoxication. Hence the stomach may, in some measure, be considered as defended from the deleterious action of the fatal dose; and this, with her having once or twice vomited copiously, may have materially assisted in saving this woman from a premature death.

The dose of arsenic which Dr. Roget's patient took was, I believe, mixed with bread and butter; but, whether the powder was strewed over the surface or previously mixed with the butter, I cannot at this moment recollect. In either case, the whole of the powder must have been effectually swallowed.

It seems to be a growing evil, the employment of arsenic for effecting suicide and murder; and frequent instances have lately occurred of horses and other animals having also been destroyed by these means. I, very lately, had occasion to analyze the contents of the stomach of a most valuable horse, which had been poisoned by arsenic; and, with some others of minor consequence, I may add the late murder perpetrated in the county of Essex, for which the culprit very deservedly suffered death. He had effected his horrid scheme by administering the powder in the form of pills, some of which were sent to me for analysis. Besides the proof of the quality of the pills, which was very palpable; the clumsy form in which they were finished shewed that no professional person had prepared them; a circumstance that still added to the veracity of the evidence. I was not subpœnaed upon that trial, there being sufficient proofs to convict the man (James Emery,) without my aid. The opinion and report I wrote and sent in to my employers, was almost verbatim the same as delivered by one or two of the witnesses; I presume, therefore, that they also had analyzed another portion of the pills, and met with the same result.

Having, in all my experiments, relied on the tests which I have so often recommended for the detection of arsenic, particularly the two *triple salts of silver*, I shall not now enter again upon that subject. All the thoughtless assertions respecting difficulties, and of the similarity of *arsenite* of silver with the *phosphate* of the same metal, are fully refuted and set aside; and most of the arguments, indeed, on both sides of the question, are to be found in several of the preceding volumes of your excellent Journal.

To those who have not been habituated to such experiments, I would inculcate the propriety and advantage to be derived from observation, by a few examples, and applying the tests to a solution of arsenic, made expressly for the purpose. I might say, that this is incumbent on all professional men; for, if we refer to the numerous trials that have occurred, and where the cause of death was from this poison, (especially one which took place in Cornwall,) we shall find that physicians, surgeons, and apothecaries, alone were taken as witnesses. Amongst other cautions which are requisite, and should always be observed, let the suspected solution be *quite cold*, when either of the ammoniacal preparations is applied; and let the experiments be always made by *day-light*.

I regret that this woman's case had not fallen into other hands, and that more attention on my part could not be bestowed to render it more complete. I visited her not more than three times, and these were not daily; so that the progressive state of the pulse and many other particulars, are evidently wanting. Should magnesia prove a useful antidote, I certainly would always prefer its *carbonate*. Indeed, on many accounts, calcined magnesia is not so good a medicine; for, in the usual process of preparing it, by submitting the carbonate to a red heat, the magnesia becomes, I may say, too pure: it loses all its water as well as the carbonic acid, and becomes a kind of anhydrate, a dry and harsh substance, divested of all moisture whatever; justifying the well-known chemical axiom, "*corpora non agunt nisi soluta sint.*"

*Long-Acre; Nov. 13th,* 1821.

